# The differences in molecular profiles and survival outcomes between early-onset and late-onset glioblastoma multiforme

**DOI:** 10.1016/j.gendis.2025.101638

**Published:** 2025-04-11

**Authors:** Yukui Wei, Xiaolong Wu, Hua Bao, Yao Xiao, Huantong Diao, Siheng Liu, Bingyang Shan, Peng Ding, Ye Cheng, Xinru Xiao

**Affiliations:** a Department of Neurosurgery, Xuanwu Hospital, Capital Medical University, Beijing 100032, China; b Geneseeq Research Institute, Nanjing Geneseeq Technology Inc., Nanjing, Jiangsu 210000, China; c Department of Neurosurgery, The First Affiliated Hospital of Kunming Medical University, Kunming, Yunnan 650032, China

Glioblastoma multiforme (GBM), defined as *IDH*-wild-type diffuse gliomas (WHO CNS grade IV), is the most common and aggressive primary brain tumor.^1^ Age significantly influences GBM, with mutational landscape varied by age.^2^ One of the most significant updates in the 2021 WHO classification is the division of diffuse gliomas into pediatric-type and adult-type gliomas, reflecting the growing understanding of their distinct molecular drivers and prognostic implications.^1^ Low-grade gliomas are more common in children, with only a 7% rate of malignant transformation from low-grade gliomas, while adults are more commonly linked to high-grade gliomas and have a transformation rate of at least 50%, resulting in a higher incidence of secondary GBM. Compared with adult GBM, pediatric GBM primarily shows *PDGFRA* amplification, lacks *EGFR* amplification, *PTEN*, and *IDH1* hotspot mutations, and has a higher frequency of chromosome 1q gain along with lower frequencies of chromosome 7 gain and 10 loss.^3^ Despite significant advancements in GBM research, studies with large cohorts examining the impact of age on mutational profiles and clinical characteristics in adult-type GBM remain limited. In this study, we conducted an in-depth analysis of the characteristics of early-onset and late-onset GBM using a large-scale in-house GBM cohort alongside the GBM cohort from The Cancer Genome Atlas (TCGA) database.

A total of 139 patients admitted to the participating Xuanwu Hospital, Capital Medical University, and diagnosed with GBM between January 2019 and April 2024 were screened, and only adult patients with available clinical records were enrolled in this study. Among these patients, 11 patients were classified as early-onset GBM (age ≤40), and 128 patients were with late-onset GBM (age >40; Fig. 1), stratified by the age cutoff of 40 years in accordance with previous literature.^4^ There are no significant differences in sex distribution and family cancer history between early-onset and late-onset GBM patients (Table S1). Pre-treatment tumor samples were collected and underwent targeted next-generation sequencing of 425 cancer-related genes using the GeneseeqPrime™ panel (Nanjing Geneseeq Technology Inc., Nanjing, China). This study was approved by the Medical Ethics Committee of Nanjing Geneseeq Medical Laboratory (NSJB-MEC-2024-08), and written informed consent from all patients was collected. We also identified the clinical data and molecular mutation profiles of GBM patients from the TCGA database by consulting previously literature,^5^ including age, overall survival, survival status, *TERT* promoter mutations, chromosome *1p/19q* codeletion, chromosome 7 gain and 10 loss, *MGMT* promoter methylation, and *BRAF* V600E mutation. The patients with incomplete age or survival data were excluded. The final sample size was 589 GBM patients, including 76 early-onset and 513 late-onset GBM patients. The study workflow schema is shown in Figure 1A.

**Figure 1:**
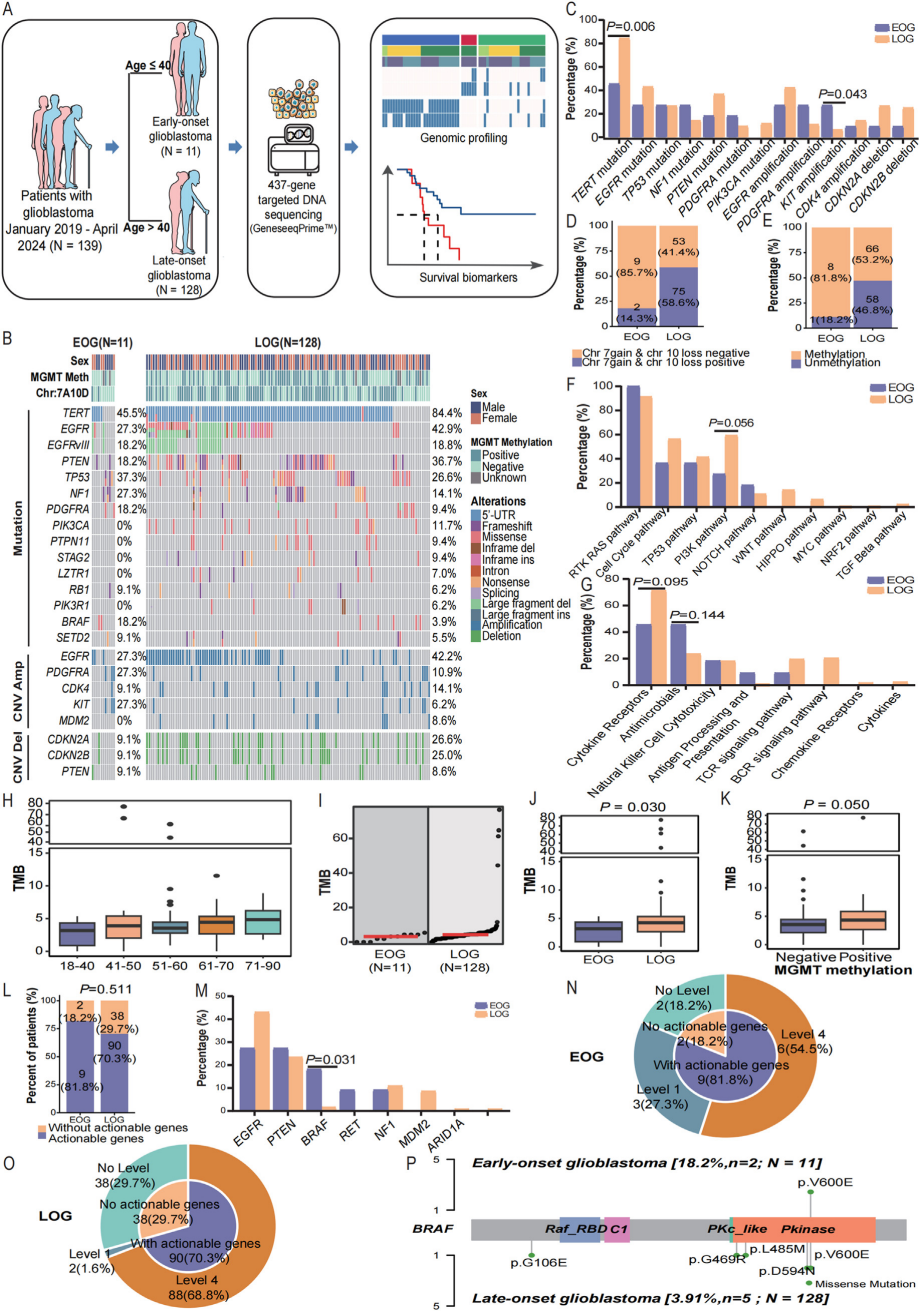
Clinical characteristics and mutation landscapes between early-onset and late-onset glioblastoma. **(A)** The study workflow schema. **(B)** Oncoprint of molecular landscapes of patients with early-onset and late-onset glioblastoma. **(C)** Bar plot of mutation and copy number variation frequencies in early-onset and late-onset glioblastoma. **(D, E)** Bar plot of chromosome 7 gain/10 loss and MGMT promoter methylation frequencies in early-onset and late-onset glioblastoma. **(F, G)** Bar plot of tumor and immune-related signaling pathway alteration frequencies in early-onset and late-onset glioblastoma. **(H)** Box plot of the distribution of tumor mutation burden (TMB) across different age intervals within the study cohort. **(I, J)** Dot plot and box plot of tumor mutation burdens in early-onset and late-onset glioblastoma. **(K)** Box plot of tumor mutation burdens in MGMT methylation positive and negative patients. **(L)** Stacked bar plot of percentage of early-onset and late-onset glioblastoma patients carrying actionable mutations. **(M)** Bar plot of actionable mutation frequencies in early-onset and late-onset glioblastoma. **(N, O)** Pie charts of actionable mutation levels in early-onset and late-onset glioblastoma. **(P)** Lollipop plot of BRAF mutation loci in early-onset and late-onset glioblastoma. EOG, early-onset glioblastoma; LOG, late-onset glioblastoma.

In the TCGA database, patients with late-onset GBM exhibit significantly poorer outcomes compared with those with early-onset GBM (Fig. S1A). The *TERT* promoter mutation, chromosome 7 gain, and chromosome 10 loss are more prevalent in late-onset GBM (Fig. S1B, C). The percentages of other molecular mutations show no significant differences between early-onset and late-onset GBM patients (Fig. S1D–F).

The mutational profiles of early-onset and late-onset GBM are displayed in Figure 1B. The most frequently mutated genes in early-onset GBM are *TERT* (45.5%), *TP53* (37.3%), and *EGFR* (27.3%), whereas in late-onset GBM, the most mutated genes are *TERT* (84.4%), *EGFR* (42.9%), and *PTEN* (36.7%; Fig. 1B). The most prevalent copy number variations in both early-onset and late-onset GBM are *EGFR* amplification and *CDKN2A/B* deletion (Fig. 1B).

The molecular landscapes of early-onset and late-onset GBM were compared. *TERT* mutations, particularly promoter alterations, were more prevalent in late-onset GBM, whereas *KIT* amplifications occurred more frequently in early-onset GBM patients (Fig. 1C). Interestingly, patients with late-onset GBM more frequently exhibit chromosome 7 gain and chromosome 10 loss (7A10D; *P* = 0.012) as well as positive *O*^6^-methylguanine-DNA methyltransferase (*MGMT*) promoter methylation (*P* = 0.043), while chromosome *1p/19q* co-deletion, microsatellite stability, and tumor mutation burden remain comparable (Table S1 and Fig. 1D, E). Concerning tumor and immune-related signaling pathways, the *PI3K* (*P* = 0.056) and cytokine receptor pathways (*P* = 0.095) exhibited marginally higher alteration frequencies in patients with late-onset GBM (Fig. 1F).

It is noted that tumor mutation burden in GBM increases with age (Fig. 1H, I) and is significantly higher in late-onset patients (*P* = 0.030; Fig. 1G). Patients with positive *MGMT* promoter methylation status shows higher tumor mutation burden values (*P* = 0.050; Fig. 1K); however, no differences in tumor mutation burden distribution were observed among patients with early-onset and late-onset GBM categorized by these factors (*MGMT* promoter methylation, chromosome 7 gain and 10 loss, *KIT* amplifications, and *TERT* mutation) (Fig. S2A–D).

The percentage of patients with actionable mutations is similar (*P* = 0.511) between early-onset (9/11, 81.8%) and late-onset GBM (90/128, 70.3%; Fig. 1L and Table S2), as defined by the OncoKB database (https://www.oncokb.org/actionable-genes). Notably, patients with early-onset GBM have a significantly higher frequency of *BRAF* mutations compared with those with late-onset GBM (*P* = 0.031; Fig. 1M). Additionally, although the overall percentage of actionable mutations is similar between the two groups, early-onset GBM patients predominantly have level 1 actionable mutations (3/11, 27.3%; Fig. 1N), whereas late-onset GBM patients are more likely to have level 4 actionable mutations (88/128, 68.8%; Fig. 1O). Among early-onset GBM patients with *BRAF* mutations, both are identified with the *BRAF* V600E mutation. In contrast, *BRAF*-mutated late-onset patients show greater diversity, with only 40% (2/5) being identified with the *BRAF* V600E mutation (Fig. 1P).

In summary, this study comprehensively explored the differences in molecular profiles and outcomes between early-onset and late-onset GBM patients. Our findings suggest that late-onset GBM patients have a poorer prognosis, marked by negative biomarkers like *TERT* promoter mutations, chromosome 7 gain, and chromosome 10 loss, though they exhibit higher *MGMT* methylation frequencies, indicating a greater likelihood of benefiting from temozolomide chemotherapy, while early-onset GBM patients show higher *BRAF* mutation frequencies, indicating a greater potential response to *BRAF* inhibitors.

## CRediT authorship contribution statement

**Yukui Wei:** Writing – original draft, Formal analysis, Data curation. **Xiaolong Wu:** Writing – original draft, Visualization, Methodology, Formal analysis, Data curation. **Hua Bao:** Formal analysis, Data curation. **Yao Xiao:** Formal analysis, Data curation. **Huantong Diao:** Formal analysis, Data curation. **Siheng Liu:** Formal analysis, Data curation. **Bingyang Shan:** Formal analysis, Data curation. **Peng Ding:** Writing – review & editing, Conceptualization. **Ye Cheng:** Writing – review & editing, Conceptualization. **Xinru Xiao:** Writing – review & editing, Conceptualization.

## Ethics declaration

The study was reviewed and approved by the Medical Ethics Committee of Nanjing Geneseeq Medical Laboratory (NSJB-MEC-2024-08), and written informed consent from all patients was collected.

## Data availability

The datasets used and/or analyzed during the current study are available from the corresponding author upon reasonable request.

## Funding

This work is supported by the 10.13039/501100001809National Natural Science Foundation of China (No. 82373403 to Y.C.), the Association Foundation Program of Yunnan Provincial Science and Technology Department and Kunming Medical University (China) (No. 202001AY070001-160 to P.D.), and the Special Grant for High-level Personnel of Yunnan Province, China (No. L-2019020 to P.D.)

## Conflict of interests

Jiaohui Pang, Hua Bao, and Yao Xiao are employees of Nanjing Geneseeq Technology Inc. All other authors declared no conflict of interests.
