# Supplementary material for: The differences in molecular profiles and survival outcomes between early-onset and late-onset glioblastoma multiforme
Source: Genes Dis. 2025 Apr 11;13(2):101638. doi: 10.1016/j.gendis.2025.101638 (PMC12664586; doi:10.1016/j.gendis.2025.101638)
Supplement: Multimedia component 1 [file mmc1.docx]

**Supplementary Material**


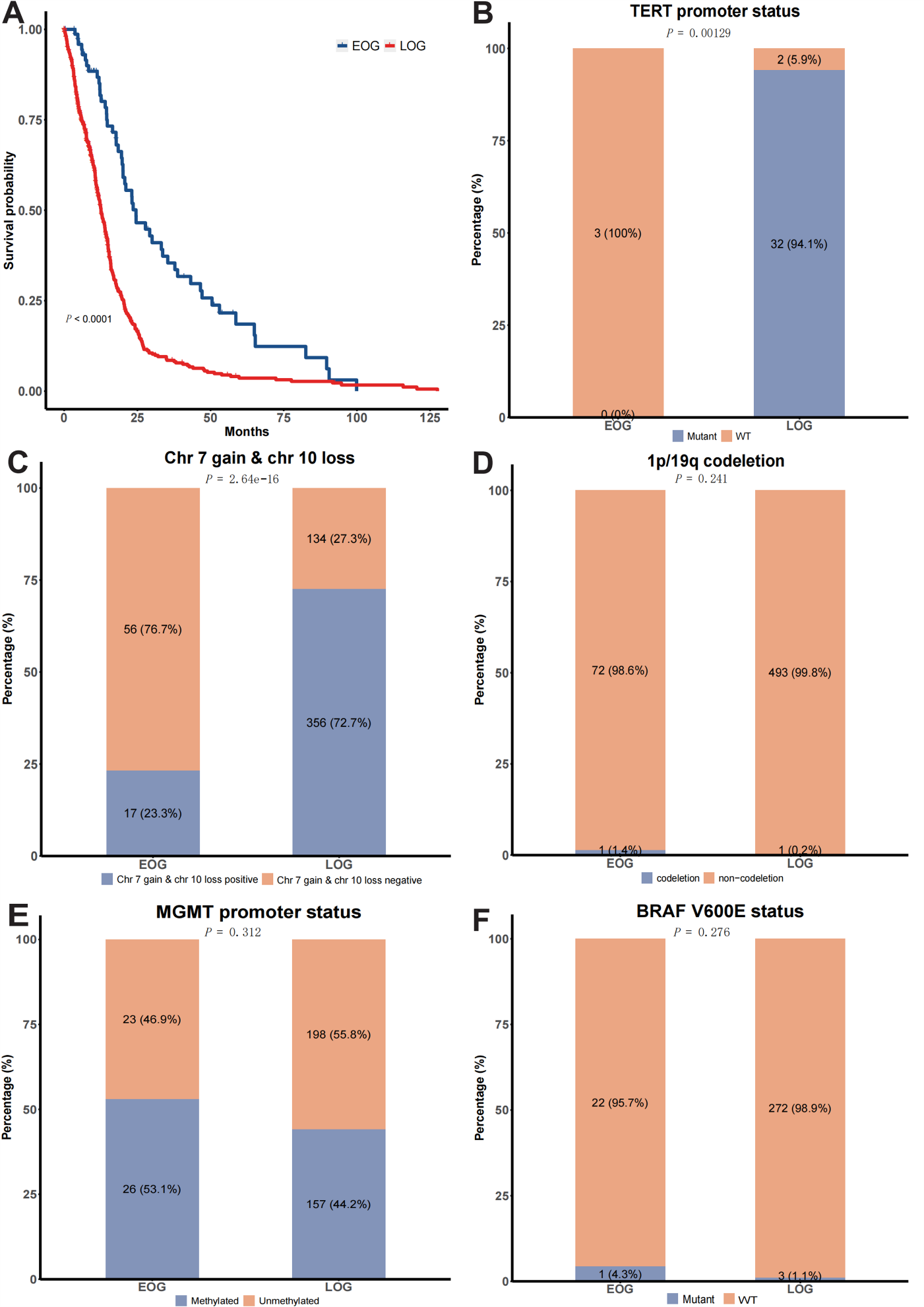


**Fig. S1** Survival and molecular mutation differences between early-onset and late-onset glioblastoma (TCGA). (A) Kaplan-Meier estimates of overall survival of patients with early and late-onset glioblastoma; (B-F) The percentages of TERT promoter mutation, chromosome 7 gain and chromosome 10 loss, chromosome 1p/19q co-deletion, MGMT promoter methylation and BRAF V600E mutation between early-onset glioma and late-onset glioblastoma.

Note: EOG, early-onset glioblastoma; LOG, late-onset glioblastoma

**
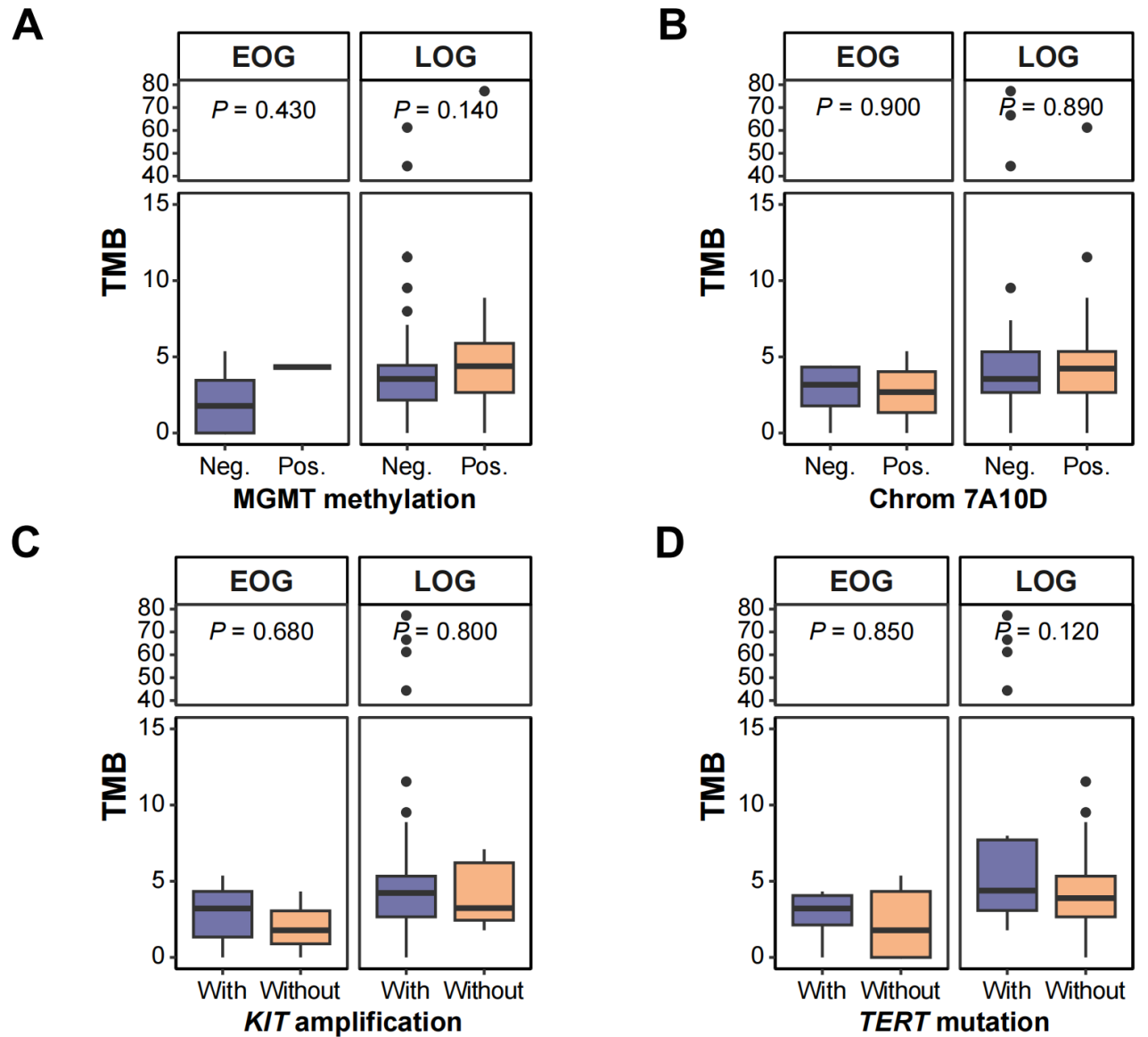
**

**Fig. S2** Comparisons of tumor mutation burdens (TMB) in patients with different molecular characteristics. (A-D) Box plots depicting the TMB distribution differences among patients with early-onset and late-onset glioblastoma categorized by (A) *MGMT* methylation status, (B) chromosome 7 gain and chromosome 10 loss, (C) *KIT* amplification status, and (D) *TERT* mutation status.

Note: EOG, early-onset glioblastoma; LOG, late-onset glioblastoma.

**Table S1: Clinical and molecular characteristics of the study cohort.**

| **Characteristics** | **EOG, No.(%)**  **N=11** | **LOG, No.(%)**  **N=128** | ***P**** |
| --- | --- | --- | --- |
| **Sex** |  |  | >0.999 |
| Female  Male | 5(45.5%) | 60(46.9%) |  |
|  | 6(54.5%) | 68(53.1%) |  |
| **Age** |  |  |  |
| Median(min-max) | 32(18-40) | 59(42-86) |  |
| **Family history of cancer** |  |  | 0.365 |
| No | 11(100%) | 108(84.4%) |  |
| Yes | 0 | 20(15.6%) |  |
| **Family history of gliomas** |  |  | >0.999 |
| No | 11(100%) | 127(99.2%) |  |
| Yes | 0 | 1(0.8%) |  |
| **Chrom 1p19q codel** |  |  | >0.999 |
| Negative  Unknown | 9(100%) | 125(100%) |  |
|  | 2 | 3 |  |
| **Chromosome 7A10D** |  |  | **0.012** |
| Negative | 9(85.7%) | 53(41.4%) |  |
| Positive | 2(14.3%) | 75(58.6%) |  |
| **Methylation of MGMT** |  |  | **0.043** |
| Negative  Positive  Unknown | 8(81.8%) | 66(53.2%) |  |
|  | 1(18.2%) | 58(46.8%) |  |
|  | 2 | 4 |  |
| **Microsatellite instability status** |  |  | >0.999 |
| MSS/MSI-L | 11(100%) | 124(98.4%) |  |
| MSI-H | 0 | 2(1.6%) |  |
| Unknown | 0 | 2 |  |
| **Tumor mutation burden** |  |  | >0.999 |
| <10muts/Mb | 11(100%) | 123(96.1%) |  |
| ≥10muts/Mb | 0 | 5(3.9%) |  |

* P values are calculated using Fisher’s exact test.

EOG, early-onset glioblastoma; LOG, late-onset glioblastoma

**Table S2: Actionable genetic alterations in patients with early-onset and late-onset glioblastoma.**

| **Groups** | **OncoKB levels** | **Genes** | **Actionable genetic alterations** | **No.(%)** |
| --- | --- | --- | --- | --- |
| **EOG** | Level1 | *BRAF* | V600E | 2(18.2%) |
|  | Level1 | *RET* | Fusions | 1(9.1%) |
|  | Level4 | *PTEN* | Oncogenic Mutations | 2(18.2%) |
|  | Level4 | *EGFR* | A289V/Amplification | 2(18.2%) |
|  | Level4 | *MDM2* | Amplification | 1(9.1%) |
|  | Level4 | *NF1* | Oncogenic Mutations | 1(9.1%) |
|  | No Level | No | No | 2(18.2%) |
| **LOG** | Level1 | *BRAF* | V600E | 2(1.6%) |
|  | Level4 | *MDM2* | Amplification | 9(7.0%) |
|  | Level4 | *NF1* | Oncogenic Mutations | 6(4.7%) |
|  | Level4 | *EGFR* | A289V/R108K/Amplification | 54(42.2%) |
|  | Level4 | *CDKN2A* | Oncogenic Mutations | 1(0.8%) |
|  | Level4 | *ARID1A* | Truncating Mutations | 1(0.8%) |
|  | Level4 | *PTEN* | Oncogenic Mutations | 17(13.3%) |
|  | No Level | No | No | 38(29.7%) |

EOG, early-onset glioblastoma; LOG, late-onset glioblastoma

**Materials and Methods**

**Patient enrollment and sample collection**

A total of 139 patients admitted to the participating Xuanwu Hospital, Capital Medical University and diagnosed with glioblastoma between January 2019 and April 2024 were screened, and only adult patients with available clinical records were enrolled in this study. Pre-treatment tumor samples were collected and underwent targeted next-generation sequencing (NGS) of 425 cancer-related genes using the GeneseeqPrime^TM^ panel (Nanjing Geneseeq Technology Inc., Nanjing, China). This study was approved by the Medical Ethics Committee of Nanjing Geneseeq Medical Laboratory (NSJB-MEC-2024-08) and written informed consents from all patients were collected. The study workflow schema is shown in Figure 1.

**The Cancer Genome Atlas validation cohort**

We identified the clinical data and molecular mutation profiles of glioblastoma patients from the TCGA database by consulting previously literature(1), including age, OS, survival status, *TERT* promoter mutations, chromosome *1p/19q* codeletion, chromosome 7 gain and 10 loss, *MGMT* promoter methylation and *BRAF* V600E mutation. The patients with incomplete age or survival data were excluded. The final sample size was 589 glioblastoma patients, including 76 early and 513 late-onset patients.

**DNA extraction, library preparation, and targeted enrichment**

DNA extraction, library preparation, and sequencing were performed at a CAP/CLIA-accredited clinical testing laboratory (Nanjing Geneseeq Technology Inc). Formalin-fixed paraffin-embedded (FFPE) samples underwent deparaffinization using xylene, followed by genomic DNA extraction with the QIAamp DNA FFPE Tissue Kit (Qiagen, Hilden, Germany), according to the manufacturer's protocol. Genomic DNA from white blood cell sediments was extracted as the normal control using the DNeasy Blood and Tissue Kit (Qiagen, Hilden, Germany). DNA quality and quantity were evaluated using the Nanodrop2000 (Thermo Fisher Scientific, Waltham, MA, USA) and the Qubit 4.0 fluorometer (Life Technology, Waltham, MA, USA), respectively. Next-generation sequencing (NGS) libraries were prepared with the KAPA Hyper Prep Kit (KAPA Biosystems, Wilmington, MA, USA), and target enrichment was performed using the GeneseeqPrime™ targeted NGS panel and the xGen Lock-down Hybridization and Wash Reagents Kit (Integrated DNA Technologies, Coralville, IA, USA). Finally, the enriched libraries were sequenced on the Illumina Hiseq4000 platform, adhering to the manufacturer's guidelines.

**Variant calling, tumor mutation burden calculation, and copy number variation identification**

The sequencing data were demultiplexed and processed for quality control of FASTQ files using Trimmomatic. Low quality reads (quality score < 15) and N based were filtered from further analysis. Qualified reads were aligned to the human reference genome (hg19) using the Burrows-Wheeler Aligner (BWA-MEM). Subsequent base quality score recalibration and local realignment around insertions and deletions (indels) were conducted with the Genome Analysis Toolkit (GATK). Duplicate reads were removed using Picard.

Single-nucleotide variants (SNVs) and indels were identified using VarScan2, with SNVs exhibiting a variant allele frequency (VAF) below 1% being filtered out. Common SNVs present in more than 1% of the population, as reported in the 1000 Genomes Project and the Exome Aggregation Consortium (ExAC) database, were excluded. To further refine the mutation list, an in-house list of recurrent artifacts was applied based on a pool of normal whole blood samples. Parallel sequencing of matched white blood cells was performed for each patient to eliminate sequencing artifacts, germline variants, and clonal hematopoiesis.

The criteria for identifying somatic SNVs and indels in tumor samples included altered reads ≥ 3, VAF ≥ 1%, and sequencing depth ≥ 30X. Fusions were detected using DELLY, with a minimum requirement of three split reads. All identified mutations and fusions were manually validated using the Integrative Genomics Viewer (IGV).

Copy number variants (CNVs) were analyzed using CNVkit, with ploidy-adjusted copy number values. The thresholds for CNV detection were set at a fold change ≥ 1.6 for gains and ≤ 0.6 for losses. Chromosome arm-level somatic CNV calling and tumor mutation burden (TMB) calculation were performed according to methods detailed in prior studies(2). Microsatellite instability (MSI) status was determined based on the stability of microsatellite loci covered by the GeneseeqPrime™ panel, with samples classified as MSI if more than 40% of the loci were found to be unstable(3).

**Signaling pathway analysis**

For signaling pathway analysis, genetic alterations were categorized based on previously established oncogenic signaling pathways from The Cancer Genome Atlas (TCGA)(4), as well as immune-related pathways identified on the ImmPort platform (https://www.immport.org/shared/)(5). Patients with mutations in any pathway-related gene were classified as positive. Gene Set Enrichment Analysis (GSEA)(6) was performed to assess and compare pathway activity differences between early-onset and late-onset groups using the clusterProfiler, ReactomePA, and ggplot2 packages in R (version 4.1.2).

***MGMT* promoter methylation**

*MGMT* promoter methylation was evaluated using droplet digital PCR (ddPCR) according to a previously established protocol(7). Genomic DNA from tissue samples was subjected to bisulfite conversion with the EZ DNA Methylation-Gold kit (Zymo Research, USA), following the manufacturer's instructions. Custom-designed primers and probes targeting the *MGMT* promoter were utilized for ddPCR, enabling the simultaneous detection of both methylated and unmethylated DNA strands. Fluorescence signals generated during ddPCR indicated the presence of methylation, with positive droplets representing methylated DNA.

**Statistical analysis**

Categorical variables between two groups were compared using Fisher’s exact test, while the distribution of continuous variables was assessed with the Wilcoxon rank-sum test. To evaluate trends in ranked data across ordered groups, we applied the Kruskal-Wallis test for trend. Kaplan-Meier survival curves were employed to visualize survival differences among patient subgroups, with statistical significance determined by the log-rank test. Hazard ratios (HR) and 95% confidence intervals (CI) were calculated using Cox proportional hazards regression analysis. A two-sided P-value of less than 0.05 was considered statistically significant. All statistical analyses were conducted using R version 4.1.2 (R Foundation for Statistical Computing, Vienna, Austria). Graphs were generated with GraphPad Prism version 8.0.1 (GraphPad Software, California, USA) and R version 4.1.2.

**Reference:**

1. Ceccarelli M, Barthel FP, Malta TM, Sabedot TS, Salama SR, Murray BA, et al. Molecular Profiling Reveals Biologically Discrete Subsets and Pathways of Progression in Diffuse Glioma. Cell. 2016;164(3):550-63.

2. Fang W, Ma Y, Yin JC, Hong S, Zhou H, Wang A, et al. Comprehensive Genomic Profiling Identifies Novel Genetic Predictors of Response to Anti-PD-(L)1 Therapies in Non-Small Cell Lung Cancer. Clin Cancer Res. 2019;25(16):5015-26.

3. Zhang X, Liu F, Bao H, Wang A, Han M, Wu X, et al. Distinct genomic profile in h. pylori-associated gastric cancer. Cancer Med. 2021;10(7):2461-9.

4. Sanchez-Vega F, Mina M, Armenia J, Chatila WK, Luna A, La KC, et al. Oncogenic Signaling Pathways in The Cancer Genome Atlas. Cell. 2018;173(2):321-37 e10.

5. Bhattacharya S, Andorf S, Gomes L, Dunn P, Schaefer H, Pontius J, et al. ImmPort: disseminating data to the public for the future of immunology. Immunol Res. 2014;58(2-3):234-9.

6. Subramanian A, Kuehn H, Gould J, Tamayo P, Mesirov JP. GSEA-P: a desktop application for Gene Set Enrichment Analysis. Bioinformatics. 2007;23(23):3251-3.

7. Abarna R, J R, Chacko G, Pai R. Droplet digital PCR (ddPCR) using FFPE DNA to assess methylation status of MGMT gene among patients with IDH mutant astrocytoma and IDH wild-type glioblastoma. J Clin Pathol. 2023;76(12):860-4.
